# Hospital Utilization, Treatment Modalities, and Mortality Using Different Biopsy Methods in Infants With Biliary Atresia

**DOI:** 10.7759/cureus.24726

**Published:** 2022-05-04

**Authors:** Anmol Mittal, Aaron Kahlam, Alexander Le, Sushil Ahlawat, Iona M Monteiro

**Affiliations:** 1 Internal Medicine, Rutgers University New Jersey Medical School, Newark, USA; 2 Gastroenterology and Hepatology, Rutgers University, Newark, USA; 3 Pediatric Gastroenterology, Rutgers University New Jersey Medical School, Newark, USA

**Keywords:** liver biopsy, healthcare utilization, national inpatient sample, biopsy method, biliary atresia

## Abstract

Objectives

To present a nationwide retrospective analysis of the sequelae and aftereffects of different liver biopsy methods in the care of pediatric patients with biliary atresia.

Methods

The National Inpatient Sample 2001-2013 database was queried for a primary diagnosis of biliary atresia and stratified based on biopsy type including percutaneous, surgical, laparoscopic, and transjugular. Patient demographics, length of stay, hospital costs, type of treatment, and mortality were compared by biopsy type. One-way analysis of variance test and multivariable logistic regression were used for analysis with α < 0.05.

Results

A total of 4,306 patients with biliary atresia were identified, of whom 2,293 underwent no biopsy, and 723 and 1,080 underwent a percutaneous or surgical biopsy, respectively. Significant differences in socio-demographics were demonstrated between the biopsy types. The length of stay and hospital charges were statistically significantly different between the biopsy types where patients without biopsies had the smallest length compared to percutaneous, surgical, and combination of biopsies. Overall, the Kasai procedure was done more frequently compared to direct liver transplantation, and compared to other biopsy types, undergoing a combination of biopsies had the highest odds of undergoing either procedure.

Conclusions

When comparing different biopsy methods, surgical biopsies of the liver outperformed percutaneous biopsies in hospital utilization and progression to definitive treatments with the Kasai procedure. Our research indicated that vulnerable populations such as minorities or the indigent may undergo inferior treatments or infrequently undergo definitive treatment. The need for definitive diagnostic guidelines is understated in patients with biliary atresia.

## Introduction

Cholestasis in the newborn, defined as a serum direct (conjugated) bilirubin > 1 mg/dL and >20% of the total serum bilirubin, affects one in 2,500 infants [1-3]. While the most common cause of cholestasis in the newborn is biliary atresia, the differential is broad [4,5]. Some conditions can be managed nonoperatively, but others require urgent surgical intervention. It is well known that the presence of cholestatic jaundice equivalates to hepatic dysfunction and is always detrimental [4]. As biliary atresia is high on the differential (being one of the most prevalent causes in the neonate), a liver biopsy is an important diagnostic and prognostic step, with greater than 90% accuracy, for patients with biliary atresia [6-8]. Current established guidelines recommend the evaluation of a cholestatic infant with an accurate history and physical examination, followed by the proper diagnostic evaluation. In the diagnostic evaluation, first and foremost is the evaluation of the liver's synthetic and biochemical function looking at the aspartate aminotransferase, alanine aminotransferase, gamma-glutamyl transpeptidase, alkaline phosphatase, and internationalized normalized ratio. The next step in evaluation consists of diagnostic imaging including abdominal ultrasound and hepatobiliary scintigraphy [4]. Though these imaging modalities are recommended, there does not appear to be a benefit in elucidating the etiology of the cholestasis as biliary atresia is unable to be differentiated from other diseases. Thus, the liver biopsy remains the most important part of the evaluation to evaluate for biliary atresia [9-11].

Liver biopsies represent a very small portion of the entire liver, even in a neonate; therefore, there is a high risk for sampling error, especially based on the type of biopsy [12]. There are multiple modalities for liver biopsy to choose from, i.e., surgical, percutaneous, or transjugular. Surgical liver biopsies are typically performed either open or laparoscopically, allowing for direct visualization of the liver to obtain a larger sample. However, it requires patients to be in the operating room utilizing anesthesia [13-15]. Percutaneous liver biopsies are less expensive and less invasive and can be done either blind utilizing a percussion-guided approach or with ultrasound guidance via a suction needle. Lastly, the transjugular approach is used in patients with coagulopathies and a high risk of bleeding and can have inconsistent samples, so they are rarely done [13,14,16]. Though there is a movement in pediatric gastroenterology to find alternative methods for diagnosing other cholestatic diseases, the ability to identify biliary atresia with a high specificity remains a challenge. Reviewing the literature, there is a significant lack of research understanding factors guiding the physician's choice regarding biopsy methods. Though physician skill and personal preference cannot be overlooked, guidelines addressing appropriate biopsy methods may help ease the burden of physician choice. We aim to analyze healthcare utilization costs, length of stay, and mortality along with socio-demographic factors in infants who undergo different types of liver biopsy to better guide physician decision-making. We also look further at how these factors impact definitive treatment options with the liver transplant or Kasai’s procedure.

This article was previously presented as a meeting abstract at the 2020 American Association for the Study of Liver Diseases, The Liver Meeting, Digital Experience on November 13-16, 2020.

## Materials and methods

Data source/study population

We used the National Inpatient Sample (NIS) 2001-2013 database to retrospectively identify patients with a primary diagnosis of biliary atresia. As a 20% sample of the United States population, NIS has been utilized for understanding healthcare utilization of disease and procedures. This database was queried for patients with biliary atresia using International Classification of Diseases, Ninth Revision (ICD-9) code 751.61 in the first three diagnosis codes indicating primary admission diagnosis. Percutaneous, surgical, laparoscopic, and transjugular biopsies were identified with ICD-9 procedure codes along with liver transplant and the Kasai procedure (Table 1) [17]. This was a retrospective study that did not involve active recruitment or enrollment of patients. As such, informed consent was not required by the institutional review boards.

**Table 1:** International Classification of Diseases, Ninth Revision (ICD-9) diagnostic and procedure codes

ICD-9 diagnostic and procedure codes	
ICD-9 diagnostic code	Diagnosis
751.61	Biliary atresia
ICD-9 procedure code	Procedure description
50.11	Percutaneous biopsy
50.12	Surgical (open) biopsy
50.13	Transjugular biopsy
50.14	Laparoscopic biopsy
50.19	Other diagnostic biopsy (accounted for as laparoscopic)
50.5	Liver transplant
50.51	Auxiliary liver transplant
50.59	Other transplant of liver
51.37	Kasai procedure (anastomosis of hepatic duct to gastrointestinal tract)

Study variables/outcome

Only patients aged six months (183 days) or below with biliary atresia were categorized into groups based on the type of biopsy they underwent: no biopsy, percutaneous, surgical, laparoscopic, transjugular, or a combination of the biopsies. After patients were stratified, patient demographics were evaluated for differences correlated with the type of biopsy performed. Length of stay and hospital costs were analyzed. Liver transplantation and Kasai procedures based on biopsy type and odds of each procedure, controlling for socio-demographic variables, were studied. Finally, mortality rates from different biopsies were compared.

Patient characteristics and demographics

Patient demographics including age, race, gender, median household income, and insurance status were gathered. Patient age in days, as reported by the NIS database, was assessed with respect to the date of the qualifying liver biopsy. To estimate national trends, estimated patient discharge weights provided by the NIS project were used.

Statistical analysis

Utilizing the weighting method for national estimates, all analyses were adjusted appropriately. A one-way analysis of variance (ANOVA) was conducted to evaluate the null hypothesis of no difference in the hospital burden and length of stay based on the type of biopsy done. ANOVA testing was also performed to compare mortality outcomes based on the different biopsy types. A chi-square analysis was performed to evaluate variables to be included in the regression analysis. Multivariable logistic regression was performed to examine demographic and social variables. All tests were performed with a significance level of α < 0.05.

## Results

We identified 4,306 infants diagnosed with biliary atresia from 2001 to 2013 who were under the age of six months (183 days). Of patients, 2,293 did not undergo a biopsy, 723 underwent a percutaneous biopsy, 1,080 underwent a surgical open biopsy, 15 underwent a laparoscopic biopsy, and none underwent a transjugular biopsy. A total of 195 patients underwent a combination of biopsies (Table 2). From 2001 to 2006, the ICD-9 codes referred to laparoscopic biopsies as “other” and were accounted for in our research as such. The average age for patients undergoing no biopsies was older (93 days), compared to those undergoing percutaneous biopsies (69 days), surgical biopsies (65 days), laparoscopic biopsies (67 days), and combined biopsies (62 days). Due to the small sample size, transjugular and laparoscopic biopsies were not accounted for in the regression analysis or the ANOVA.

**Table 2:** Patient data by biopsy (N) * Significant level p < 0.05.

Variable	No biopsy (2,293)		Percutaneous biopsy (723)		Surgical biopsy (1,080)		Transjugular biopsy (0)		Laparoscopic biopsy (15)					Combined biopsy (195)	P-value			
Age (mean days old)	93		69		65		-		67					62	<0.001>			
Race															<0.002>			
Caucasian	986		307		405		-		5					89				
African American	437		132		228		-		0					23				
Hispanic	459		139		232		-		0					49				
Asian, Pacific Islander, Native American	411		145		215		-		10					34				
Median income															<0.001>			
0-25th percentile	595		140		286		-		5					39				
26-50th percentile	446		170		277		-		5					69				
51-75th percentile	719		146		258		-		5					41				
76-100th percentile	533		267		259		-		0					46				
Sex															0.004*			
Males	1,077		376		464		-		5					93				
Females	1,216		347		616		-		10					102				
Insurance cohorts															<0.001>			
Private insurance	966		308		482		-		5					82				
Medicaid	1,176		362		547		-		10					93				
Medicare	0		5		0		-		0					0				
No insurance	151		48		51		-		0					20				
Liver transplant															0.637			
No transplantation	2,240		704		1,062		-		15					190				
Transplantation	53		19		18		-		0					4				
Kasai procedure															<0.001>			
No procedure	2,014		512		386		-		5					65				
Procedure	279		211		694		-		10					130				
Mortality															0.009*			
Alive	2,269		718		1,080		-		15					195				
Died	24		5		0		-		0					0				

Multivariable logistic regression analysis for biopsies demonstrated that compared to patients without biopsies, those with percutaneous biopsies were more likely performed for those in the fourth median income quartile (OR: 1.64) (Table 3). All other attributes were not significant. Of the patient populations, African Americans compared to Caucasians and those with Medicaid compared to private insurance were less likely to get open surgical biopsies (OR: 0.77 and 0.83, respectively). As compared to no biopsy, patients of the female gender were less likely to get a combination of biopsies as compared to their male counterparts (0.76). Those in the second and fourth median income quartiles were more likely to get a combination of therapies compared to the first median income quartile (OR: 1.60 and 1.83, respectively). All other categories were not statistically significant for differences in biopsy type and demographics.

**Table 3:** Multinomial logistic regression comparing biopsy types * Significant level p < 0.05.

	Percutaneous biopsies vs. no biopsies (reference)			Surgical (open) biopsies vs. no biopsies (reference)					Combination biopsies vs. no biopsies (reference)		
Variable	P-value	Odds ratio (95% CI)		P-value		Odds ratio (95% CI)			P-value	Odds ratio (95% CI)	
Race											
Caucasian	Reference			Reference					Reference		
African American	0.082	1.29 (0.97-1.71)		0.009^*^		1.40 (1.09-1.80)			0.101	0.62 (0.34-1.10)	
Hispanic	0.282	1.17 (0.88-1.55)		0.007^*^		1.42 (1.10-1.83)			0.132	1.40 (0.90-2.16)	
Asian, Pacific Islander, Native American	0.061	1.30 (0.99-1.70)		0.031^*^		1.32 (1.03-1.70)			0.747	1.08 (0.68-1.72)	
Gender											
Males	Reference			Reference					Reference		
Females	0.091	0.84 (0.69-1.03)		0.001^*^		1.45 (1.21-1.74)			0.152	0.78 (0.56-1.10)	
Insurance status											
Private insurance	Reference			Reference					Reference		
Medicaid	0.924	1.01 (0.81-1.27)		0.155		0.86 (0.70-1.06)			0.421	1.18 (0.79-1.75)	
Medicare	0.987	0.00 (0.00-0.00)		1.00		0.00 (0.00-0.00)			1.00	0.00 (0.00-0.00)	
No insurance	0.001^*^	4.87 (2.14-11.08)		0.290		0.80 (0.53-1.21)			0.007^*^	4.83 (1.55-15.07)	
Median Income											
0-25th percentile	Reference			Reference					Reference		
26-50th percentile	0.001^*^	1.70 (1.25-2.31)		0.003^*^		1.48 (1.14-1.92)			0.001^*^	2.86 (1.79-4.57)	
51-75th percentile	0.955	1.01 (0.76-1.35)		0.048^*^		0.78 (0.61-0.99)			0.463	0.82 (0.49-1.39)	
76-100th percentile	0.001^*^	2.10 (1.58-2.80)		0.862		1.02 (0.79-1.33)			0.061	1.63 (0.98-2.70)	
Mortality											
Alive	Reference			Reference					Reference		
Died	0.852	1.11 (0.39-3.16)		0.953		0.86 (0.70-1.06)			0.976	0.00 (0.00-0.00)	

Comparing the length of stay for the different types of biopsies, it was seen that patients who did not have biopsies stayed for the least number of days (m = 8.56) as compared to those undergoing a percutaneous biopsy (m = 10.82), an open surgical biopsy (m = 11.80), or a combination of biopsies (19.40). ANOVA testing demonstrated a significant (p < 0.001) difference in the length of stay.

Comparing the hospital costs, it was seen that patients who did not have biopsies performed had the least hospital charge (m = $48,073) as compared to those with a percutaneous biopsy (m = $65,728), an open surgical biopsy (m = $57,387), or a combination of biopsies (m = $102,776). ANOVA testing demonstrated a significant difference in hospital costs.

The mortality rate for the overall population was estimated to be around 0.66%. For patients who underwent no biopsy, the rate was 1.03%. Patients who underwent a percutaneous biopsy had a rate of 0.69%. Patients undergoing transjugular and laparoscopic biopsies did not have any mortalities reported. Patients with a surgical biopsy had a mortality rate of 0%. Finally, those who underwent a combination of procedures also had a mortality rate of 0%. ANOVA testing demonstrated a nonsignificant difference in the mean mortality rates of different biopsy types.

The overall rate for liver transplants was estimated to be 2.20%. After performing a logistic regression (Table 4), it was found that females were less likely to undergo liver transplantation, though not statistically significant (OR: 0.81). Compared to Caucasians, Asians/Native Americans/Pacific Islanders and Hispanics were more likely to undergo liver transplantation (OR: 1.97 and 2.03, respectively). When looking at median income quartiles, it was found that compared to the first, the third and fourth quartiles were more likely to undergo transplantation (OR: 2.63 and 3.47, respectively). When looking at insurance status, compared to private insurance, other insurance statuses were not significantly more or less likely to undergo a liver transplant. For patients who underwent no biopsy, 2.29% received transplants. Of patients undergoing percutaneous biopsies, 2.69% had a liver transplant. Of patients undergoing surgical open biopsies, 1.69% had a liver transplant and 2.29% of patients with a combination of biopsies had one. ANOVA testing demonstrated a nonsignificant difference in the rates of liver transplants based on biopsy type.

**Table 4:** Socio-demographic predictors of liver transplant * Significant level p < 0.05.

Variable	P-value	Odds ratio (95% CI)
Race		
Caucasian	Reference	
African American	0.336	0.63 (0.24-1.62)
Hispanic	0.029^*^	2.03 (1.08-3.81)
Asian, Pacific Islander, Native American	0.026^*^	1.97 (1.08-3.60)
Gender		
Males	Reference	
Females	0.813	0.81 (0.50-1.32)
Insurance status		
Private insurance	Reference	
Medicaid	0.788	0.93 (0.52-1.64)
Medicare	0.999	0.00 (0.00-0.00)
No insurance	0.998	0.00 (0.00-0.00)
Median income		
0-25th percentile	Reference	
26-50th percentile	0.652	0.78 (0.26-2.34)
51-75th percentile	0.016^*^	2.63 (1.20-5.76)
76-100th percentile	0.002^*^	3.47 (1.57-7.65)
Biopsy type		
No biopsy	Reference	
Percutaneous biopsy	0.999	1.00 (0.54-1.87)
Surgical biopsy	0.748	1.09 (0.62-1.95)
Combined biopsy	0.995	0.00 (0.00-0.00)

The rate of undergoing the Kasai procedure in the patient population was 30.73%. After performing a logistic regression (Table 5), it was found that females were more likely to undergo the procedure (OR: 1.13), though not statistically significant. Compared to Caucasians, Hispanics were less likely to undergo the Kasai procedure (OR: 0.59). When looking at median income quartiles, it was found that compared to the first, the second quartile was more likely to undergo the procedure (OR: 1.36). Though it was not statistically significant, the third quartile was found to have an increased odds of undergoing the procedure while the fourth quartile was less likely. When looking at insurance status, compared to private insurance, Medicaid and uninsured patients were less likely to get the Kasai procedure (OR: 0.90 and 0.38, respectively). Of patients with no liver biopsy performed, 12.17% underwent the procedure. Of patients that had a percutaneous biopsy done, 29.15% also had the Kasai procedure and were more likely than non-biopsy patients to get one (OR: 3.07). A total of 64.23% of patients who had a surgical open biopsy underwent the procedure as well. Of patients undergoing a combination of biopsies, 66.63% also underwent the Kasai procedure. Both of these groups were more likely than non-biopsy patients to get a liver transplant (OR: 10.41 and 18.74, respectively). ANOVA testing demonstrated a significant difference in the rates of the Kasai procedure based on biopsy type.

**Table 5:** Socio-demographic predictors of Kasai procedure * Significant level p < 0.05.

Variable	P-value	Odds ratio (95% CI)
Race		
Caucasian	Reference	
African American	0.144	0.83 (0.64-1.07)
Hispanic	0.000^*^	0.59 (0.45-0.76)
Asian, Pacific Islander, Native American	0.844	1.03 (0.80-1.31)
Gender		
Males	Reference	
Females	0.192	1.13 (0.94-1.35)
Insurance status		
Private insurance	Reference	
Medicaid	0.012^*^	0.90 (0.78-1.02)
Medicare	0.999	0.00 (0.00-0.00)
No insurance	0.016^*^	0.38 (0.17-0.83)
Median income		
0-25th percentile	Reference	
26-50th percentile	0.022^*^	1.36 (1.04-1.77)
51-75th percentile	0.302	1.14 (0.89-1.46)
76-100th percentile	0.116	0.81 (0.62-1.05)
Biopsy type		
No biopsy	Reference	
Percutaneous biopsy	0.000^*^	3.07 (2.42-3.88)
Surgical biopsy	0.000^*^	10.41 (8.41-12.88)
Combined biopsy	0.000^*^	18.74 (12.76-27.53)

## Discussion

Cholestasis of the newborn is a common finding that carries a wide differential [4,5]. Liver biopsy remains a key diagnostic step in the pathway to treatment and therefore understanding the risks and benefits of the different biopsy methods is important for physicians to decide the next best step for their patients. A reassuring finding in our study was evidence that there was no increase in mortality rates in patients undergoing biopsies compared to their counterparts. The lack of improvement in mortality rate is thought to be due to the cross-sectional nature of our study, an accurate extended analysis would better establish the long-term benefits of undergoing diagnostic biopsies. Our results showed that likely due to its invasive nature, patients undergoing surgical biopsy stayed one more day in the hospital; however, they had a lower total hospitalization cost compared to patients who had a percutaneous biopsy. Though demonstrated to be safer in the current literature, our study demonstrates a higher significant odd of all non-Caucasian races undergoing a surgical biopsy compared to other biopsy types. In our patient population, insurance status played a significant role, as those infants without insurance were much more likely to undergo a percutaneous biopsy or a combination of the two biopsies versus a surgical biopsy. Finally, income appeared to play an inconsistent role in determining the type of biopsy being performed as the 26-50th quartile was more likely to get either type of biopsy compared to the 0-25th, but the 51-75th quartile was less likely to get either type of biopsy.

Percutaneous liver biopsies in children have been shown to be generally safer compared to surgical biopsies, which seemingly contradicts our results, as there appeared to be no change in mortality rates between the biopsy types. The North American Society for Pediatric Gastroenterology, Hepatology, and Nutrition guidelines recommend that a percutaneous liver biopsy be performed in most infants with undiagnosed cholestasis [18]. While percutaneous biopsy appears to be generally safe, biliary atresia tends to affect a very young population that has unique risks, which may result in increased hospital burden. One possible reason for this contradiction may be that younger age has been shown to be associated with an increased risk of bleeding following percutaneous biopsy [19-21]. During a surgical biopsy, bleeding can be identified and addressed intraoperatively preventing further complications [13]. Furthermore, laparoscopic biopsies are minimally invasive and allow for direct observation of the biliary tree, as well as the opportunity to address additional findings intraoperatively [22]. With fewer complications, and the ability to respond and adjust to new information in real time, a surgical biopsy can save several diagnostic steps, resulting in decreased hospital burden while providing an adequate diagnostic sample. Further research into the effect of age on complications of percutaneous biopsy may clarify and provide support for our findings.

Despite the decreased hospital burden in surgical biopsies, our results demonstrated that there was an increase in the length of stay in surgical patients. This can likely be attributed to the possible extra time required post-operatively to observe the infant compared to their percutaneous counterparts. Few studies have analyzed differences in diagnosis and outcomes in patients with biliary atresia by race and insurance status. Harpavat et al. showed that non-Hispanic blacks and Hispanics were likely to be referred later in their disease course compared to their Caucasian counterparts, which likely resulted in worse disease at the time of treatment [23].

While our study demonstrated no significant trends with respect to liver transplants, there was a significant role of socio-demographics in predicting the performance of the Kasai procedure. The current standard of care algorithm typically places the Kasai procedure as the first step and the liver transplantation as the second; therefore, due to the cutoff of six months, we likely did not capture the patient population at high risk of transplantation. Compared to the Caucasian group, the minority populations were at high risk of not undergoing the Kasai procedure. A study by Apfeld et al. showed that African Americans undergo the Kasai procedure at a later age, which attenuates our results of this vulnerable population undergoing the procedure [24]. Those without private insurance were also less likely to undergo the procedure, the odds being 62% less in those without insurance. This demonstrates significant bias in the healthcare system against such vulnerable patients. Finally, it was demonstrated that undergoing a surgical biopsy or a combined biopsy significantly increased the odds of undergoing the Kasai procedure compared to not undergoing any biopsies pointing to the superior diagnostic capabilities of this method.

A lack of guidelines on the biopsy approach for the diagnosis of biliary atresia may mean that workup is ultimately determined by perceived cost, which our study demonstrates may not be as clearly defined. Additionally, jaundice is more difficult to detect in patients with darker skin, resulting in a more cautious and less invasive approach to diagnosis. Finally, while the role of implicit bias has not been studied in biliary atresia, it has been studied in the pediatric population overall [25-27]. Implicit bias may play a role in the diagnostic approach for biliary atresia in different populations. It is important that specific guidelines are developed in the diagnostic workup for biliary atresia to address observed disparities.

There are important limitations to consider in this study. First, a percutaneous biopsy may be performed with or without ultrasound guidance, which may affect outcomes. For the purposes of this study, both methods were considered equivalent. They may even be performed in the outpatient setting, which may attribute to the larger portion of our patient population not undergoing a diagnostic procedure during hospitalization. Further research comparing image-guided percutaneous biopsies to surgical biopsies may demonstrate that the percutaneous option is the better approach. Additionally, our analysis relies on accurate coding of diseases and procedures, which may not always be the case. Finally, the NIS database only captures data related to one hospital stay and diagnoses and procedures occurring only during that hospital stay, it does not reflect outcomes beyond the initial stay.

## Conclusions

The length of stay for biliary atresia patients who underwent different biopsy types was significantly different. Patients who underwent a percutaneous biopsy had a lower length of stay compared to those with a surgical biopsy, and both were lower than those who had a combined biopsy. Patients who underwent a percutaneous biopsy had a higher hospital burden compared to those who had a surgical biopsy but had a lower burden than those who had a combination of biopsies. Socio-demographics played a considerable role in the odds of undergoing a biopsy and specifically which type of biopsy. Socio-demographics also was a large factor in patients undergoing definitive treatment with the Kasai procedure. These unexpected results may provide additional considerations when deciding between biopsy types and may provide the call to action for creating or clarifying current guidelines.
